# The Relationship between Waist Circumference and Work-related Injury in Reference to the Fourth Korea National Health and Nutrition Examination Survey

**DOI:** 10.1186/2052-4374-25-29

**Published:** 2013-11-01

**Authors:** Sung-Kyung Kim, Ji-Min Son, Jae-Young So, Hyocher Kim, Kyungsuk Lee, Sung-Soo Oh, Sang Baek Ko

**Affiliations:** 1 Department of Occupational and Environmental Medicine, Wonju Severance Christian’s Hospital, Yonsei University, Wonju, Korea; 2 National Academy of Agricultural Science (NAAS), Rural Development Administration (RDA), Seoul, Suwon, Korea

**Keywords:** Work-related injury, Waist circumference, Risk factors

## Abstract

**Objective:**

This study aims to investigate the relationship between waist circumference and work-related injury in reference to the fourth Korea National Health and Nutrition Examination Survey.

**Methods:**

By analyzing data from the fourth Korea National Health and Nutrition Examination Survey conducted from 2007 to 2009, we estimated the rate of injury experience according to socioeconomic status, including occupational property, of 8,261 subjects. We performed logistic regression analysis with work-related injury experience rate as dependent variable and waist circumference as an independent variable, Odds ratios (OR) were calculated, which reflect the likelihood of work-related injury experience rate, and 95% confidence interval (95% CI) while controlling for relevant covariates with stratifying by sex, age, nature of injury, site of injury and occupational group.

**Results:**

Among 797 persons who had injury experience over the past 1 year, 293 persons (36.8%) had work-related injury experience. After adjusting the confounding variables, the work-related injury was related to abnormal waist circumference (OR = 1.35; 95% CI: 1.02 ~ 1.78). In subgroups, ORs were higher in men (OR = 1.42; 95% CI: 1.02 ~ 1.98), professional, manager, and administrator (OR = 2.41; 95% CI: 1.10 ~ 5.28). Higher rate of injuries were noted in back and waist (OR = 2.92; 95% CI: 1.49 ~ 5.73), and transport accident had increased risk (OR = 1.60; 95% CI: 1.13 ~ 2.28).

**Conclusions:**

Work-related injury rate differed depending on the waist circumference. The abdominal obesity was associated with higher risk of work-related injury. This study would be useful in selecting appropriate priorities for work-related injury management in Korea.

## Introduction

Based on 2011 department of Korea statistics, generalized improvement in nutrient intake by Korean population has raised the rate of obesity. The rate was found to be higher in men (36.5%) than women (26.5%) [1]. Obesity has a well-known association to raise the prevalence of cardiovascular disease or type 2 diabetes [2,3]. Also, it is known that obese person is more likely to have sprain or dislocation injury than non-obese person [4], and risk of fracture is affected by the obesity. For a person with body mass index (BMI) between 25 and 29.9 kg/m2, the fracture risk is 1.29 times higher than a person with BMI of 29.9 kg/m2. People with BMI of between 30 and 34.9 kg/m2 and between 35 and 39.9 kg/m2 had increased risks of 1.67 and 1.94 times, respectively, revealing significant increase in fracture risk with higher BMI [5].

Injury is disability of body caused by external factors, and injuries originate during work is defined as work-related injury [6]. In Korea, Industrial Accident Compensation Insurance Act states that occupational casualty is any injury, disease, disability, or death of a worker from causes originating or relating to his or her occupation [7]. These casualties are attributed by accidents or noxious agents including physical factors, chemical materials, dusts, pathogens, works that put excessive strain to body. Conventionally, exposure to physical and chemical harmful agents and unsafe behavior are explained to be major sources of work-related injury [8,9]. Occupation injury leads to pathologic condition, disability, and early mortality, and in turn, these losses translate into loss of life expectancy and increased cost in a society [10,11]. Since 2001, applicant to occupational health and safety insurance had become widespread. The number of work places that had applied for the insurance has increased by 41.7% in 2009 from 2001, and 23.8% increase in full-time workers was noted during the same period. However, these years had shown steady increase of injured workers. For work-related accident, less mortality became evident in recent years, but the number of injured persons increased every year. Rate for work place accident momentarily had increased from 2003 to 2004, but in general, the figure has stagnated or decreased at around 0.7% [12]. Injury is no longer recognized as random and unavoidable accident, but its perception has become preventable accident. Thus several nations are attempt in to set up an effectual policy for reduction of the injury, and collecting injury-related data to produce base reference in developing a policy is identified as an important agenda [13]. It is known that work-related injury and disease originate through complex interaction of several risk factors [14-16].

Recently, some claimed that obesity function as a protective factor in injury [17], but obesity is generally considered as a risk factor [18]. Body mass index (BMI) was used in several studies to define obesity. However, the index has been acknowledged inappropriate to reflect fluctuation of body fat distribution and different characteristics in obesity among individuals and population groups. In some cases, BMI is mainly attained from records that subjects had recorded for themselves rather than measuring the index for examination. This lowers validity of the data and showed inconsistency of outcomes [19,20]. Because measuring abdominal fat is known to have more sensitivity in detecting individual differences than BMI, it is more useful to discover risk factors of obesity-related disease [19,21].

There have been studies to identify obesity’s relationship to injury in a work environment, and they reported that obese persons have about 26 to 107% more incidence of injury during working than people with normal weight [14,22-24]. They in addition noted that higher BMI showed increased frequency of acute traumatic injury at work [23,25]. However, these studies used BMI only to investigate the relationship until now. As mentioned above, waist circumference has become recognized as a more useful index than BMI to study health problem associated with obesity [19,21,26], but few reports have been made about relationship between work-related injury and obesity using waist circumference. In addition, it is still unclear about abdominal obesity’s mechanism and extent to injury. Thus, it necessitates investigation and discussion about its role as a factor that incites injury [27].

In this study, its purpose is to determine relationship between waist circumference and work-related injury. The authors want to apply outcomes found in this study to manage obesity for prevention of injury at work.

## Materials and methods

### Study subjects

The Korea National Health and Nutrition Examination Survey has been performed to identify Korean health and nutrition state since 1998. The fourth Korea National Health and Nutrition Examination Survey was conducted for 3 years from 2007 to 2009 with rolling survey sampling method. Each rolling sample was extracted from the survey, and it was designed to have independent and identical properties among samples. Year-round survey (50 weeks per year) was conducted, and 200 to 250 subjects were surveyed in a week. In each year, probability samples reflecting the whole country were extracted through stratified sampling in 3 stages. First stage involved stratification of 29 strata with a reference to 11 areas’ population ratio by sex and age groups, and the samples were collected according to towns. Second stage extracted samples at district level, and final stage collected samples in family level. Number of participation to the survey was 23,632 (74.5%) among 31,705 national wide subjects [28]. Final analysis was done to 8,261 adults between 20 and 65 years old who had an experience of injury in his or her job.

### Study methods

In this study, work-related injury was defined as an experience of injury for the past 1 year from the time of the fourth Korea National Health and Nutrition Examination Survey. Those who had answered yes to a question "have you received a treatment in a hospital or emergency room due to injury or intoxication?" were reviewed, and among them people who answered yes to "during work" in additional information section were finally defined as a work-related injury experiment [29].

As demographic and socioeconomic characteristics, age, sex, waist circumference, alcohol intake, smoking state, exercise level, marital status, annual household income, and educational level were identified. Also, mechanism of injury and injury site were noted. We organized occupations into 6 groups according to major categorizations of the '6th Korean Standard Classification of Occupations’; 'manager, professional, and administrator’, 'clerk’, 'service and sales worker’, 'skilled agricultural, forestry, and fishery worker’, 'craft, equipment, machine operating, and assembling worker’, and 'elementary worker’ [29]. Abdominal obesity is termed for man with waist circumference of 90 cm or above and woman with the circumference of 85 cm or above [30].

In this study, injury rates were calculated according to demographic and occupational characteristics of study subjects. Also, injury experience was set as a dependent variable, and abdominal obesity was used as an independent variable. Using the variables, logistic regression analysis was performed to calculate corrected odds ratio (OR) of work-related injury experience to population and 95% confidence interval (CI). Stratified analysis was carried out for age, sex, injury mechanism, injury site, and occupational groups. SAS 9.2 program (SAS Inc., Cary, NC, USA) was used for statistical analysis, and statistical significance was determined at 0.05 or below.

## Results

### General and occupational characteristics of study subjects

Table 1 listed general and occupational characteristics of study subjects. Male consisted 55.4% of total subjects, and age with 40 or above accounted 58.8%. Waist circumference was within the normal range for 6,093 subjects (74.1%) and above the normal range for 2,179 subjects (25.9%). For male, there were 27.7% of study subjects who had waist circumference of 90 cm or above, and for female, the proportion with abnormal waist circumference was 24.7%. Percentage of people who had suffered an injury over the 1 year was 7.2%, and 3.3% of study subjects had sustained their injuries from work. There were 23% of population working in 'service and sales worker’ group, and next was followed by 'manager, professional, and administrator’ group with 20.9%, 'craft, equipment, machine operating, and assembling worker’ group had 18% of population working for the sector, Proportions for 'elementary worker’ group and 'clerk’ group were 13.7% and 13.6%, respectively. 'Skilled agricultural, forestry, and fishery worker’ group had the least figure of 10.8% working in the sector. For injury mechanism, motor vehicle accident (43.3%) was found to be the most common cause. Fall, slip down, and collision were found in 35.4%, and laceration, amputation, stab, and injured by machine were identified in 14.4% as their injury mechanisms, Burn and intoxication were answered by 3.4% of study subjects. Unknown and other causes were noted in 3.4%. Subjects had fairly even distribution on sites of their injuries. Head, face, and neck were injured in 24.5%. Back and waist were affected in 27.5%, and upper and lower extremities were answered as their injury site in 23.8% and 21.8%, respectively.

**Table 1:** General characteristics of participants

**Variables**		**Prevalence (%)**
Sex	Male	55.4
	Female	44.6
Age	20 ~ 40y	41.2
	41 ~ 65y	58.8
Waist circumference	Male < 90 cm	72.3
	Male ≥ 90 cm	27.7
	Female < 85 cm	75.3
	Female ≥ 85 cm	24.7
Alcohol consumption (frequency)	<1 per month	26.7
	1 ~ 4 per month	36.5
	≥2 per week	36.8
Present smoking status	Smoker	28.4
	Former smoker	21.0
	Never-smoker	50.6
Family income (quartile)	Low	10.1
	Low-moderate	25.0
	Moderate-high	1.2
	High	33.7
Types of occupation	Manager, professional and administrator	20.9
	Clerk	13.6
	Service and sales worker	23.0
	Skilled agricultural, forestry, and fishery worker	10.8
	Craft, equipment, machine operating, and assembling worker	18.0
	Elementary worker	13.7
Injury experience	Yes	7.2
	No	92.8
Work-related injury experience	Yes	3.3
	No	96.7
Mechanisms of work-related injury	Transport accident	43.3
	Fall down, slip down, and collision	35.4
	Laceration, amputation, stab, and machinery accident	14.4
	Burn and poisoning	3.4
	Etc.	3.4
Location of injury	Face, neck, and head	24.5
	Back and waist	27.5
	Upper extremity	23.8
	Lower extremity	21.8
	Etc.	2.4
Marital status	Married, living	77.0
	Married, separated	1.8
	Bereavement	2.7
	Divorce	3.6
	Single	14.5
	Unknown	0.3
Exercise status	≥Moderate	29.1
	<Moderate	70.9
Education status	≤Elementary	16.2
	Middle	12.6
	High	38.2
	≥University	33.0

### Risk of work-related injury regarding to waist circumference

As listed in Table 2, multiple logistic regression analysis showed that work-related injury was 1.49 times (95% CI: 1.16 ~ 1.92) higher in abnormal waist circumference group than within normal waist circumference group. After correcting confounding variables, work-related injury was discovered to 1.35 times (95% CI: 1.02 ~ 1.78) higher in the abnormal group.

**Table 2:** Associations between waist circumference and any work-related injury

	**Non-abdominal obesity**	**Crude abdominal obesity (95% CI†)**	**Adjusted‡ abdominal obesity (95% CI)**
OR*	1	1.49(1.16 ~ 1.92)	1.35(1.02 ~ 1.78)

*Odds ratio, multiple logistic regression analysis.

†95% confidence interval.

‡Adjusted for age, sex, income, education, smoking, alcohol, occupation and exercise.

### Factors related to work-related injury in abnormal waist circumference group

Stratified multiple logistic regression analysis was made on age, sex, occupational groups, injury sites, and mechanism, and male with abnormal waist circumference had 1.42 times higher risk of injury during work (95% CI: 1.02 ~ 1.98). For 'Manager, professional, and administrator’ group, the risk of work-related injury was 2.41 times higher (95% CI: 1.10 ~ 5.28) in people with abnormal waist circumference. The abnormal waist circumference group was 2.92 times higher risk of thoracic and lumbar spine injuries (95% CI: 1.49 ~ 5.73). Motor vehicle accident also showed significant increase in risk by 1.60 times (95% CI: 1.60 ~ 1.98) (Table 3).

**Table 3:** Associations between abnormal waist circumference and work-related injury of subgroups

**Variables**		OR* (95% CI)†
Sex	Male	1.42 (1.02 ~ 1.98)
	Female	1.16 (0.68 ~ 1.97)
Age	20 ~ 40 yr	1.52 (0.90 ~ 2.57)
	41 ~ 65 yr	1.26 (0.91 ~ 1.74)
Types of occupation	Manager, professional, and administrator	2.41 (1.10 ~ 5.28)
	Clerk	0.95 (0.29 ~ 3.10)
	Service and sales worker	1.15 (0.32 ~ 2.09)
	Skilled agricultural, forestry,	1.31 (0.67 ~ 2.55)
	and fishery worker	
	Craft, equipment, machine operating, and assembling worker	1.57 (0.99 ~ 2.43)
	Elementary worker	0.72 (0.31 ~ 1.67)
Sites of injury	Face	0.49 (0.11 ~ 2.27)
	Head	1.29 (0.31 ~ 5.33)
	Neck	1.39 (0.64 ~ 3.00)
	Back and waist	2.92 (1.49 ~ 5.73)
	Chest	1.79 (0.49 ~ 6.63)
	Abdomen	0.61 (0.61 ~ 6.08)
	Upper extremity	1.62 (0.96 ~ 2.73)
	Lower extremity	1.02 (0.52 ~ 2.00)
	Etc.	0.37 (0.05 ~ 3.10)
Mechanisms of injury	Transport accident	1.60 (1.13 ~ 2.28)
	Fall down, slip down, and collision	1.41 (0.97 ~ 2.05)
	Etc.	1.19 (0.70 ~ 2.02)

*Odds ratio, multiple logistic regression analysis, adjusted for age, sex, income, education, smoking, alcohol, occupation and exercise, reference group (odds ratio = 1.00) is a normal waist circumference group.

†95% confidence interval.

## Discussion

This study investigated relationship between work-related injury and waist circumference. Subjects were selected using the 4th Korea National Health and Nutrition Examination Survey, and those who had experienced an injury over 1 year period were accounted for the study. There were 7.2% of patients who suffered injury over 1 year, and injuries that were related to work was 3.3% of the population. Waist circumference was measured to each subject to evaluate obesity, 74.1% were found to be within normal range. There were 27.7% of male population who had abnormal waist circumference, and 24.7% of female subjects had abdominal obesity.

Young age, short work experience, male, improper education about health and safety, insomnia, and work stress are known to have significant relationship with work-related injury [14,17,31,32]. Recently, obesity has been recognized as a risk factor of injury [14,33]. In this study, multiple logistic regression analysis showed that age, sex, exercise, smoking, alcohol intake, marital status, and occupations had 1.35 times higher risk of work-related injury (95% CI: 1.02 ~ 1.78). Also, in the group who are male, who had abnormal waist circumference, motor vehicle accidents, and managerial and professional jobs, and whose injury sites were back and waist illustrated significant relationship with work-related injury.

This study used waist circumference instead of BMI to determine obesity, because waist circumference is suggested by several studies to more indicative of obesity than BMI [26]. However, previous studies that studied relationship between obesity and work-related injury had used BMI as index for obesity [14,33].

In addition, reports were published concerning obesity as both hazard and protective factor in work-related injury [17]. It is considered as a risk factor because over-weight affects a person’s gait and balance. These people are likely on multiple medications, and their psychotropic effects may give additional risk for injury. Also, increased prevalence of sleep apnea syndrome in obese people may cause excessive sleepiness and fatigue. For a protective factor, obesity acts as a cushion during slip down, and increased bone density in obesity reduces fracture risk [33]. However, although positive correlation was present between bone density and BMI in a study on menopausal women, the study found that visceral fat and waist circumference had a negative correlation which led to increased risk of spine fracture [34].

In this study, people with abnormal waist circumference had 1.35 time higher risk for work-related injury. When subgroups were studied, male (OR: 1.42, 95% CI: 1.02-1.98) and 'Manager, professional, and administrator’ groups (OR: 2.41, 95% CI: 1.10-5.28) showed significant relationship. Like previous studies that determined relationship between BMI and work-related injury [25], some subgroups in our study did not show any close relationship. The reason for partial relation is believed that obesity’s biophysical risk factor had worked differently in accordance to each of subgroups.

In 2011, Canadian study on relationship between obesity and work-related injury noted that sprain, back and waist, lower extremity, and fall showed correlation to injury prevalence [14]. In one study, sprain, strain, and dislocation were significantly more in obesity diagnosed people, and fall and physical overuse were identified as causes for injury [4]. In other study, BMI and work-related injury had significant relationship, and particularly, prevalence of injury in knee and lower body were higher in obese group [25]. In addition, senile with obesity had a higher risk of fracture in one study [5].

This study showed that male with abdominal obesity had a higher injury risk than female. This supports previous studies which male with obesity had higher risks for motor vehicle accident and injury than female, and in turn the result reflects that obesity works differently to development of injury according to sex [35,36]. Motor vehicle accident and injury site such as body parts above pelvic bone are already known as a risk factor for injury in obese patients. Boulanger et al. performed a cohort study on patients with blunt injury, and they found high incidence of injury due to traffic accident in people whose BMI is 30 kg/m2 and above. They also found that frequently involved injuries were rib fracture, lung contusion, pelvic bone fracture, and fractures in extremities [37]. Mock et al. reported that people with obesity had increased risk for mortality and severity of injury from motor vehicle accident and injury to chest [38]. Although this study was not able to reproduce relationship between obesity and lower body injury that showed significant relationship in other studies [33], our study revealed that work-related injury in back area increased in a group that had abnormal waist circumference by 2.92 times. This outcome reflects abdominal obesity has a possible correlation to development of back injury. Obesity-related chronic inflammatory diseases such as hypertension, diabetes, and dyslipidemia induce atherosclerotic change in spinal vessels [39], and this led to degeneration to spine [40], affecting raised susceptibility to injury [41]. Because abdominal obesity is an index that reflects obesity associated risk factors and chronic inflammatory state well [19-21], it implies that measuring waist circumference can be an useful tool to predict the injury.

Meanwhile, not all occupational groups showed significant correlation between abnormal waist circumference and work-related injury. Only 'Manager, professional, and administrator’ group showed significant increase of work-related injury risk by 2.41 times, and this suggests that injury is affected by waist circumference found in a specific occupational group. Like previous studies that discovered significant relationship between obesity and work-related injury in office workers [33], our result had an identical evidence that white collar workers were found to have significant relationship between abnormal waist circumference and work-related injury. It is possible that high incidence of sprain in white collar group may be the reason for increased number of injury [16].

One limitation in this study is that we were not able to remove recall bias, because the study is based on memory of subjects who answered the questionnaire about their experiences of work-related injury. In addition, thought a subject suffered from an injury, the subject may have been absent because he or she was admitted to a hospital during a visit by a surveyor for an interview. Other limitations include that occupational groups, injury mechanism, and site were not classified in detail and several variables are mixed together, limiting the recognition of injury’s risk factors. Also, waist circumference was divided simply into normal and abnormal, and this simplification did not allow dose-reaction relationship. Further study that verifies distribution of injury risk in accordance with each waist circumference as continuous variable is required to determine clearer relationship. In addition, the study is insufficient to prove causal relationship between the circumference and injury because it was a cross-sectional study. Therefore, a prospective cohort study with a method that can verify risk factors in detail is needed for determining causal relationship between work-related injury and abdominal obesity, and with the study, it would be able to identify risk factors such as influence of chronic diseases, sleepiness and fatigue, and physical limitations that are suggested in previous studies [33].

## Conclusions

Despite of limitations present in this study, it again showed possible connection of abdominal obesity to work-related injury. This study is significant that waist circumference rather than BMI is presented as a predictive index to work-related injury. However, additional studies are needed to identify factors in waist circumference that affect work-related injury more clearly. With further refinement and learning about the risk factors, it would be possible to raise the need for obesity management to reduce occurrence of work-related injury in a corporation or a nation.

## Competing interests

The authors declare that they have no competing interests.

## Authors’ contributions

SKK and SBK designed the research. SKK collected the data. SKK, HK and KL performed the statistical analysis. SKK, SSO, and SBK interpreted the data. SKK, JMS and JYS wrote the manuscript. All of the authors read and approved the final manuscript.
